# Optimization of Experimental Infection of the Animal Model *Galleria mellonella* Linnaeus 1758 (Lepidoptera: Pyralidae) with the Gram-Positive Bacterium *Micrococcus luteus*

**DOI:** 10.3390/insects15080618

**Published:** 2024-08-16

**Authors:** Davide Banfi, Tommaso Bianchi, Maristella Mastore, Maurizio Francesco Brivio

**Affiliations:** Laboratory of Applied Entomology and Parasitology, Department of Theoretical and Applied Sciences (DiSTA), University of Insubria, 21100 Varese, Italy; davide.banfi@uninsubria.it (D.B.); tbianchi1@studenti.uninsubria.it (T.B.); maristella.mastore@uninsubria.it (M.M.)

**Keywords:** Lepidoptera, *Galleria mellonella*, *Micrococcus luteus*, Gram positive, infection, protocol, alternative model

## Abstract

**Simple Summary:**

In recent years, the use of alternative animal models to vertebrates for the study of infectious processes and antimicrobial drug development has become a major challenge in experimentation. Insects, in particular *Galleria mellonella,* may represent a good model for preclinical studies, as their response to infections allows for the preliminary selection of molecules with biological activity in a potential sepsis event. However, discordant data are often reported in the literature, and this is often due to the different methods implemented in many laboratories. The aim of this work was therefore to develop a standard protocol for infection with a Gram-positive bacterium, as we consider it important to apply these unified methodologies in order to obtain reproducible data. Our results made it possible to define a correct growth curve of *Micrococcus luteus* and, in parallel, an infection methodology that would provide consistent and repeatable data. We are therefore confident that this work can be a support for preclinical studies on model insects, as a link between the development of new drugs and its availability for patients.

**Abstract:**

The aim of this work was to develop an experimental protocol for the infection of *Galleria mellonella* with Gram-positive bacteria. Some physiological characteristics of these insects are comparable to those of vertebrates, therefore allowing the replacement of mammals in the preclinical phases of drug development. *G. mellonella* Linnaeus 1758 (Lepidoptera: Pyralidae) is accepted as an alternative model for the study of infectious diseases. Since data on infection procedures with different bacterial strains are scarce and sometimes conflicting, also due to different and non-uniform protocols, we developed an experimental protocol that would allow for controlled and repeatable infections, using the Gram-positive bacterium GRAS (Generally Regarded As Safe) *Micrococcus luteus*. After analyzing the morphology and defining the growth rate of *M. luteus*, doses of between 10^1^ and 10^6^ CFU/larvae were administered to late-stage larvae. The survival rate of the larvae was monitored up to 7 days and the LD_50_ determined. The bacterial clearance capacity of the larvae after injection with 10^3^ and 10^5^ CFU/larvae was assessed by hemolymph bacterial load analysis. The results made it possible to define the growth curve of *M. luteus* correlated with the CFU count; based on the LD_50_ (10^3.8^ CFU/larvae) calculated on the survival of *G. mellonella*, infections were carried out to evaluate the immune efficiency of the larvae in bacterial clearance. This protocol, standardized on *G. mellonella* larvae, could provide a functional tool to study the course of bacterial infections.

## 1. Introduction

The term “animal testing” refers to procedures performed on living animals for research purposes across various fields. These procedures aim to better understand complex biological processes and assess the effectiveness of drugs and other products, such as cosmetics, household cleaners, food additives, and industrial/agrochemical products. Animal testing adheres to the principles of the 3Rs (Reduction, Refinement, and Replacement) introduced by Russell and Burch in the late 1950s [1,2]. For scientific research, common animal models include mice, rats, pigs, and other mammals [3], as well as non-human primates [4]. Despite their closer genetic similarity to humans, these models have numerous disadvantages, such as high costs for purchasing and housing, long reproductive cycles, stringent regulatory oversight, and growing bioethical concerns. In recent years, to reduce and replace the use of these animals, alternative models, such as invertebrates, particularly insects [5,6,7,8], have been explored in various fields of experimentation.

The lepidopteran *Galleria mellonella* Linnaeus 1758 (Lepidoptera: Pyralidae), also known as the greater wax moth or honeycomb moth, is an insect of Asian origin now found throughout all warm regions of the world [9]. It has become increasingly popular as a model organism in various research areas, including toxicity assays, antimicrobial drug efficacy, and the study of virulence, mechanisms, and pathogenesis [10,11,12,13].

This insect is a hive-destroying parasite of honeybees. The larvae of *G. mellonella* colonize the combs to feed on honey, pollen, and beeswax, leaving behind a trail of webs and debris [9]. Compared to traditional animal models, *G. mellonella* larvae are cheaper to obtain and maintain, requiring no special laboratory equipment. Additionally, the structure and size (1.5–2.5 cm) of the larval stages make them easy to handle during laboratory procedures. Their innate immune system has non-self-recognition and elimination mechanisms comparable to those of vertebrates [14]. Hemolymph can be easily collected and microbiologically analyzed, making these larvae particularly useful for pharmacological and microbiological research [15]. Another important feature is that the larvae can be incubated at 37 °C, although the appropriate controls are required to monitor any alterations in the immune response [16].

The larvae can be infected with defined amounts of microorganisms or drugs via injection directly into the hemocoel through the last left pro-leg. *Micrococcus luteus* (formerly *Micrococcus lysodeikticus*) is a Gram-positive/variable, aerobic, non-motile coccal saprophytic microorganism with a high G + C content. It measures 0.5 to 3.5 μm in diameter and can appear as single cocci, diplococci, tetrads, or irregular clusters. Biochemically, *M. luteus* is positive for catalase, urease, and oxidase tests, but negative for mannose, xylose, lactose, mannitol, arginine, and galactose tests [17].

These bacteria are commonly found in natural environments, such as soil and water, and are also normal inhabitants of human skin and the oropharyngeal mucosa. *M. luteus* can be considered a clinically significant opportunistic pathogen, as it has the potential to cause infections such as hepatic and brain abscesses, endocarditis, bacteremia, and septic arthritis in immunocompromised patients [18].

Since *G. mellonella* and *M. luteus* can be used as model species for studying bacterial infections, the aim of our study was to develop a protocol to standardize this experimental infection model. This protocol could be valuable for research on the immune system of this insect and for establishing a model to study antibiotic therapies against Gram-positive bacteria in vivo.

## 2. Materials and Methods

### 2.1. Chemicals and Instruments

All reagents were supplied by Merck KGaA, (Darmstadt, Germany). Instruments were supplied by Celbio Spa (Milan, Italy) and Snijders Labs (Tilburg, The Netherlands). Centrifugations were carried out with a SIGMA 1–14 (SciQuip Ltd., Newtown, UK) microcentrifuge and an Eppendorf 5804R (Eppendorf, Hamburg, Germany) centrifuge. All materials, buffers, and solutions were autoclaved or filtered through 0.22 µm Minisart filters (Sartorius, Goettingen, Germany). Optical density was determined by a Jasco V730 UV/Vis Spectrophotometer (Jasco Europe Srl, Cremella, Italy). For microscopic observations, a Zeiss Axiolab (Carl Zeiss, Oberkochen, Germany) optical microscope connected to an OPTIKA digital camera A C-HP (Optika Microscopes, Ponteranica, Italy) and a Leica SP5 Confocal microscope (Leica Microsystems, Wetzlar, Germany) were used. 

### 2.2. G. mellonella Rearing and Maintaining Conditions

Insect larvae were originally supplied by Kreca Ento-Feed (Ermelo, The Netherlands); this facility maintains its own wax moth culture without adding antimicrobial compounds or hormones to the artificial diet. 

Subsequent generations were reared according to the method of Auke W. de Jong [19]. Larvae were reared on a sterile artificial diet (9.5% rice flour, 9.5% oatmeal, 5% wheat germ, 14.8% Torula yeast, 3.9% beeswax, 24% honey, 22.6% glycerol, 10.7% tap water) and maintained at 26 °C with 60% RH (relative humidity) in the dark in an insect climatic chamber, mod. IN011 (Darwin Chambers, St. Louis, MO, USA). For all assays, only healthy late-stage larvae, characterized by a pale-yellow color, and weighing about 300–350 mg, were selected and, during treatments, the larvae were fed with the sterile diet replaced daily. Before use, larvae were conditioned at 37 °C for 24 h and sterilized with 70% ethanol.

### 2.3. Cultures of M. luteus

*M. luteus* ATCC 4698 strain (Merck KGaA, Darmstadt, Germany), in the lyophilized form, was inoculated in liquid Luria–Bertani (LB) medium and incubated at 37 °C in the dark at 180 rpm overnight in a bench shaker incubator. After overnight growth, the optical density of the culture was evaluated at 600 nm (OD_600_) by spectrophotometry. Then, after reaching an OD value of 0.60, using a sterile loop, the bacteria were smeared in 3 quadrants on tryptic soybean agar (TSA) plates, then incubated at 37 °C overnight. Before liquid culture, a single colony was characterized by Gram staining [20]. A single colony with (diameter 3.5 mm) was picked up, inoculated into fresh LB medium (20% volume of the flask), and incubated at 37 °C in an orbital shaker at 180 rpm. Culture growth was monitored hourly by spectrophotometry and by track dilution assay on TSA plates.

### 2.4. M. luteus Growth Curve and Colony Counts

To monitor bacterial growth, 100 µL of the liquid bacteria culture were drawn hourly, diluted 1:10 into sterile LB medium, and the optical density was measured by spectrophotometry at OD_600_. 

In order to determine the colony forming units (CFUs), a slightly modified track dilution assay was performed [21] over the course of 24 h. Then, every hour and after OD reading, 2 µL of culture were taken and diluted 1:100 into sterile LB medium; subsequently, serial dilutions were carried out in a 96 multi-well chamber. In detail, a 10 µL drop for each dilution was plated on a TSA Petri dish, spreading the drop by tilting the plate vertically; plates were then incubated at 22 °C (to avoid overgrowth and for easy reading) overnight. At the end of the incubation period, the CFUs were counted and only plates included in a range of up to 100 CFUs were evaluated. Finally, CFU/mL was calculated, considering the number of colonies and the relative dilution factors.

### 2.5. Infection of G. mellonella Larvae and Bacterial Clearance

For survival assays, late-stage healthy *G. mellonella* larvae were used. Three batches of 10 larvae, conditioned at 37 °C for 24 h, were surface sterilized with 70% ethanol. Before survival assays, 1 mL of *M. luteus* overnight culture was washed twice with sterile PBS and centrifuged at 1000× *g* for 20 min, to remove metabolites of the medium that could interfere with the infection. Before injection, the bacterial pellets were resuspended in sterile PBS and appropriately diluted in the range of 10^1^ up to 10^6^ CFU/larvae. Larvae were microinjected into the abdominal spiracle area with a gas tight micro syringe with adjustable volumetric doses (Hamilton, 500 µL, mod. 1750 Luer Tip Threaded Plunger Syringe) with a hypodermic needle (30G × ½”), injecting 10 µL of each bacteria dilution. Infected larvae were incubated at 37 °C, 60% RH, in the dark in a climatic chamber, and the survival was monitored up to 7 days. Survival was assessed by a stereomicroscope, evaluating both motility after stimulation with tweezers and melanization levels; from the data obtained, LD_50_ (dose which causes the death of 50% of larvae) was calculated. In order to further check the infection progress, batches of 3 larvae were microinjected with 10 µL of *M. luteus* suspension containing a dose of 10^3^ and 10^5^ CFU/larvae and incubated for 2 and 6 h at 37 °C. After incubation, the larvae were anesthetized on ice and hemolymph samples (about 100 μL/larvae) were taken by puncture of the abdominal region and collected into sterile tubes and diluted 1:10 in sterile LB medium. Finally, 100 µL of each sample were plated on TSA. Plates were then incubated at 22 °C overnight and the CFU/mL was counted. As control, hemolymph samples from PBS-injected larvae were plated. The experimental procedure is summarized in Table 1.

### 2.6. Statistical Analysis

Data are reported as mean ± standard deviation (SD). The data obtained for the different variables showed a normal distribution after the D’Agostino and Pearson’s normality test. Ten replicates were performed for the growth curve; and five replicates were performed for trace dilution tests. In survival tests, three different pools of ten larvae were analyzed and the Kaplan–Meier curve was derived; LD_50_ was calculated with a non-linear regression function implemented in GraphPad prism 9.0 software (Graph Software INC., LA Jolla, CA, USA).

## 3. Results

### 3.1. Growth Assessment of M. luteus

**Figure 1:**
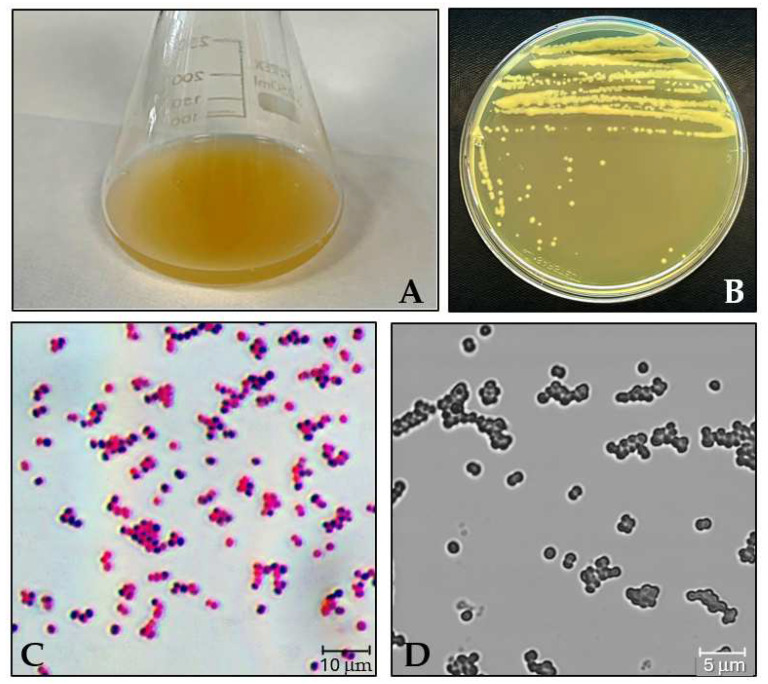
Erlenmeyer flask liquid culture of *M. luteus* in LB medium (**A**); solid culture on a TSA medium in plate (**B**); microscopy characterization and morphology of *M. luteus* by Gram staining (**C**) and confocal microscopy (**D**).

*M. luteus* was identified through liquid and solid culture, microscopic assessment, and Gram staining. In LB medium, the culture exhibited a dark-yellow color (Figure 1A), while on the solid medium, the colonies appeared as small to medium-sized, yellow, smooth, shiny, circular, and concave (Figure 1B). *M. luteus* is a Gram-positive microorganism typically arranged as singular cocci, diplococci, tetrads, or irregular clusters (Figure 1C,D).

**Figure 2:**
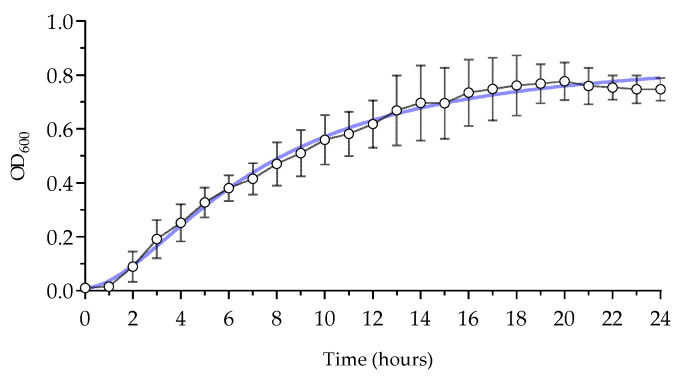
Growth curve (blue line) of *M. luteus* monitored by spectrophotometry (OD_600_ ± SD) for 24 h at 37 °C, (n = 10).

The results from the liquid growth curves are presented in the graph and table (Figure 2 and Table 2). The initial lag phase persists until the second hour of growth, followed by the onset of the exponential growth phase, which stabilizes around the 13th to 14th hour. The OD600 of the diluted culture increased from 0.01516 to approximately 0.75 after 16 h and then remained stable until the 24th hour.

**Figure 3:**
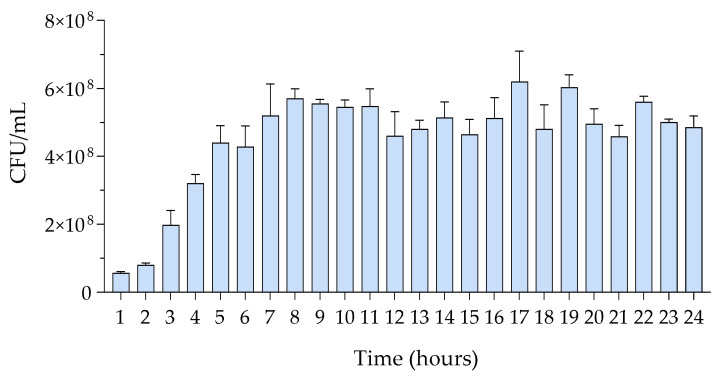
CFU/mL ± SD of *M. luteus* by means of track dilution test, (n = 5).

Track dilution tests demonstrated the relationship between exponential growth, as measured by OD600, and microbial counts. At time t = 0, a single, well-isolated bacterial colony (3.5 mm in diameter) was used for inoculation. The number of colonies per milliliter of starter culture was determined by plating different dilutions. During the exponential growth phase, the number of CFUs increased rapidly and stabilized around the 8th to 9th hour, reaching approximately 10^8^ CFU/mL. This count remained relatively stable up to 24 h (Figure 3 and Table 3).

### 3.2. Survival of G. mellonella after Infection with M. luteus

**Figure 4:**
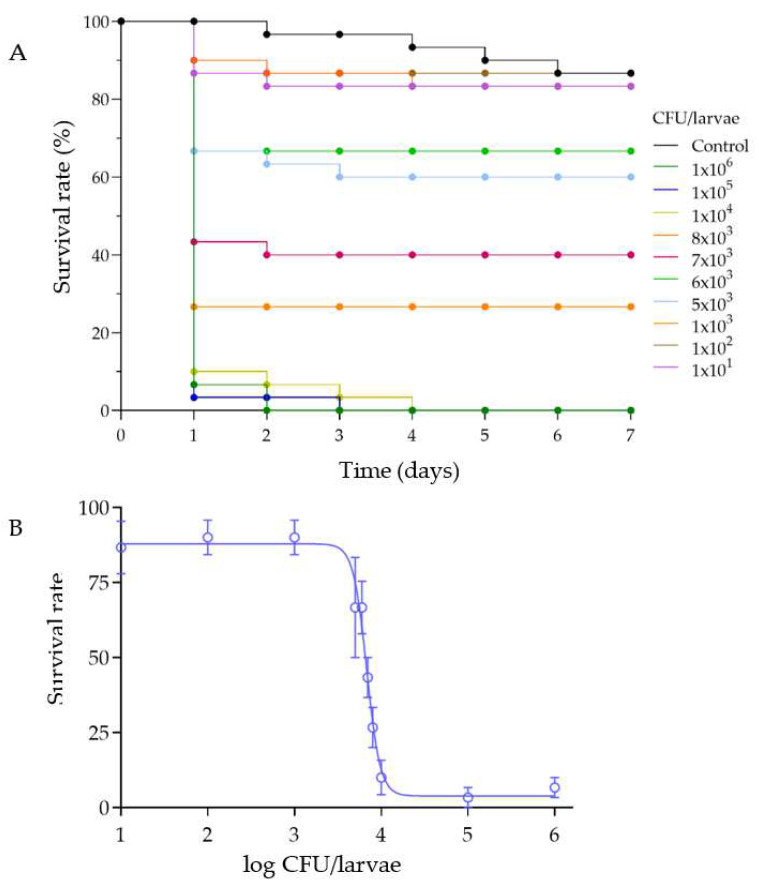
(**A**): Kaplan-Meier curves of *G. mellonella* larvae survival infected with different doses of *M. luteus*, during a 7-day period, (n = 3). (**B**): *G. mellonella* survival rate after injection with different doses of *M. luteus* over the first 24 h (n = 3).

To determine the survival rate of G. mellonella after infection, various doses of bacteria were injected into the larvae, and mortality was monitored for up to 7 days. It was observed that the highest mortality occurred within the first 24–48 h (Figure 4A). Larvae treated with different doses of *M. luteus* showed mortality rates comparable to the control (PBS-injected larvae) up to a dose of 10^3^ CFU/larvae (Figure 4B). However, larvae infected with doses ranging from 10^4^ to 10^6^ CFU/larvae exhibited complete mortality. The LD_50_ value, calculated using the nonlinear regression function, was found to be 10^3.8^ CFU/larvae.

### 3.3. Bacterial Clearance in G. mellonella Hemolymph

**Figure 5:**
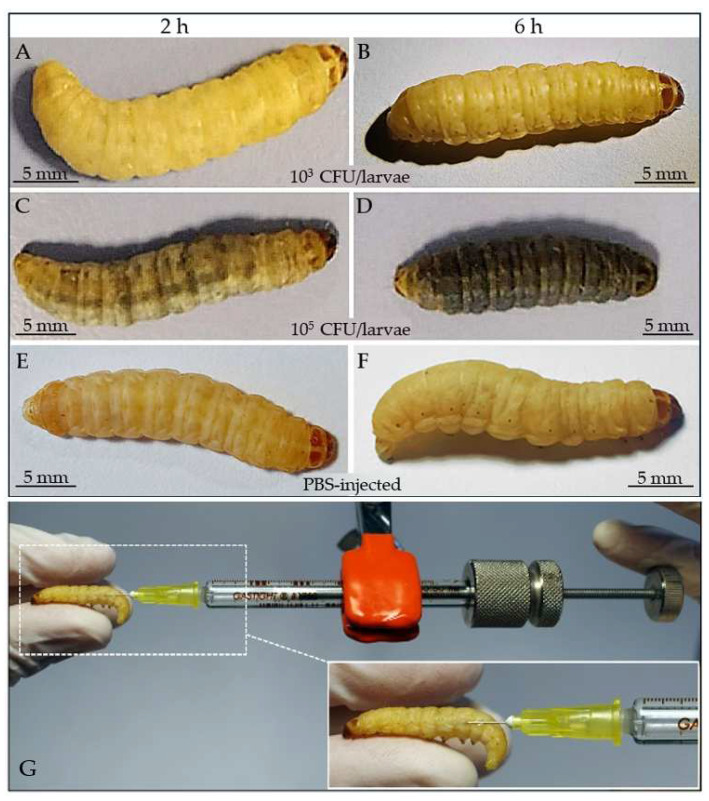
Larvae of *G. mellonella* injected with *M. luteus* 10^3^ CFU/larvae (**A**,**B**) and 10^5^ CFU/larvae (**C**,**D**). Note the darkening observed after infection with the higher dose. (**E**,**F**) show larvae injected with PBS. (**G**) shows the procedure for injecting larvae with a gas-tight Luer threaded plunger syringe.

Morphological changes after infection were assessed at different times by injecting *M. luteus* at doses of 10^3^ and 10^5^ CFU/larvae (Figure 5). At the lower bacterial dose (10^3^ CFU/larvae), the larvae exhibited no noticeable signs of melanization (dark coloring) or distress at any time point (Figure 5A,B). In contrast, significant melanization was observed in larvae injected with 10^5^ CFU/larvae at both time points (Figure 5C,D). PBS-injected larvae, used as controls, showed no melanization (Figure 5E,F).

**Figure 6:**
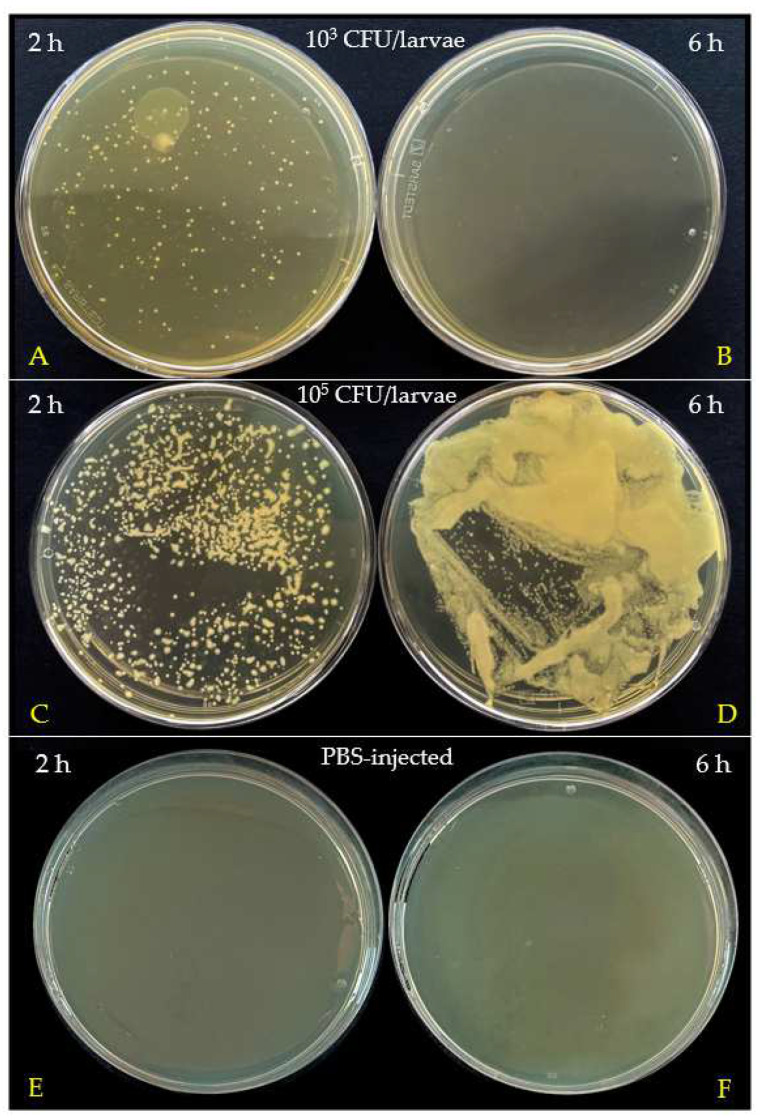
Bacterial growth in hemolymph after 2 and 6 h post infection with *M. luteus* at doses of 10^3^ CFU/larvae (**A**,**B**) and 10^5^ CFU/larvae (**C**,**D**). Controls: (**E**,**F**).

The above observations were also supported by colony counts in hemolymph. Hemolymph was collected and analyzed two and six hours post injection. Samples were diluted 1:10 in sterile LB and 100 µL were plated on TSA and finally incubated at 22 °C overnight (Figure 6).

The hemolymph collected 2 h post infection with the lower dose (10^3^ CFU/larvae) contained 2.03 × 10^4^ CFU/mL (Figure 6A). However, samples collected after 6 h showed no colonies (Figure 6B), suggesting an effective bacterial clearance by the immune system. Conversely, the hemolymph from larvae treated with the higher dose (10^5^ CFU/larvae) showed a high number of colonies (>3.73 × 10^4^ CFU/mL) at 2 h (Figure 6C). After 6 h, colonies in the hemolymph were confluent (Figure 6D), indicating that this bacterial load was not effectively cleared by the larvae immune processes. Hemolymph samples from PBS-injected larvae showed no bacterial colonies (Figure 6E,F).

## 4. Discussion

The successful development of a standardized protocol for infecting *Galleria mellonella* larvae with *Micrococcus luteus* ATCC 4698 provides a valuable model for studying infections caused by Gram-positive bacteria and evaluating antimicrobial therapies. This work aims to further validate *G. mellonella* as an alternative to mammalian models. Using this insect allows for preclinical studies while avoiding ethical, legal, and economic restrictions. Since its immune response is comparable to that of vertebrates, this model is particularly suitable for studying host–pathogen interactions [22]. As previously mentioned, *G. mellonella* offers several advantages that make the larvae particularly suitable for microbiological studies. For example, the ability to inject precise doses of bacteria directly into the hemocoel and to monitor the outcomes of infection provides a controlled environment for assessing the virulence of pathogens and the effectiveness of drug treatments [23]. Numerous studies have employed *G. mellonella* as a model for infections with Gram-positive bacteria. For instance, bacteria such as *Streptococcus pyogenes* [24,25] *Streptococcus pneumoniae* [26], *Enterococcus faecalis* [27,28,29], *Enterococcus faecium* [30,31], *Staphylococcus aureus* [32,33,34], and *Listeria monocytogenes* [35,36] have been extensively studied using this model.

Our data have shown that *M. luteus,* in both the solid and liquid cultures, exhibits consistent growth patterns, providing a solid foundation for reliable infection studies with this bacterium. The bacterial growth curve displayed an initial lag phase, followed by exponential growth, reaching a stable phase after 13–14 h. The data obtained from the growth curve are critical for determining the appropriate timing for bacterial collection to perform infection assays, ensuring consistent injections into the larvae. One of the most crucial aspects of utilizing the proposed model is the method of bacterial infection. Subcutaneous microinjection should be the preferred technique for inducing bacterial infections in *G. mellonella* larvae as it allows for precise dosage control and reliably triggers an immune response. In contrast, methods such as oral administration, though reliable, follow the intestinal route and can be influenced by the microbiota and gastric enzymes, potentially interfering with the spread of pathogens in the hemolymph. The route of bacterial administration can significantly influence experimental outcomes, as highlighted by the research conducted by Kordaczuk and colleagues [37]. Their findings demonstrated that larvae injected with 10 and 50 cells of *Pseudomonas entomophila* exhibited a dose-dependent immune response, characterized by the upregulation of immune-related genes and enhanced defense activity in the larval hemolymph. Conversely, larvae that orally ingested the pathogen showed antimicrobial activity in the hemolymph only at a dose of 10^3^ CFU/larvae, but not at 10^5^ CFU/larvae. Although the authors reported that higher doses of bacteria led to the induction of immune genes and the presence of antimicrobial peptides (AMPs) in the hemolymph, this did not result in the elimination of the bacteria.

In our study, the lethality tests indicated that *M. luteus* caused significant mortality in *G. mellonella* larvae at doses of above 10^3^ CFU/larvae, with the highest mortality observed during the first 24 h post injection. Although our data were reproducible in laboratory tests, other studies have highlighted that the responses of the larvae to infections can vary significantly depending on the bacterial strain and serotype. The LD_50_ value of 10^3.8^ CFU/larvae obtained with *M. luteus* might serve as a benchmark for studies assessing bacterial virulence or the protective efficacy of potential antimicrobial compounds. Evans and colleagues reported significant differences in the LD_50_ values following the injection with 10⁶ cells of *Streptococcus pneumoniae* strains England^14^ and Portugal^19F^, with the observed differences correlating with the presence of specific virulence factors [26]. Furthermore, the experiments conducted by Loh and colleagues [24] demonstrated that infection with the reference strain *Streptococcus pyogenes* serotype M1 strain SF370 resulted in dose-dependent larval mortality, with an LD_50_ of 6 × 10⁶ CFU/larvae. Different M serotypes (M1, M2, M3, M4, M6, M18, M28, M49) elicited a wide range of responses in *G. mellonella*, with serotype M18 being the most virulent and serotype M2 being the least. Additionally, significant differences were observed between strains carrying the M3 serotype, such as MGAS12501 and the invasive M3 strain MGAS315, with the latter showing higher mortality rates, faster melanin accumulation, and rapid hemolymph coagulation.

In some cases, discordant results have been reported even when using the same microorganism. For example, in the study by Loh and colleagues [24], a survival rate of about 90% after 24 h and 70% after 96 h was reported following infection of the larvae with 8 × 10⁶ CFU/larvae of *S. pyogenes* serotype M3 strain MGAS315. In contrast, Olsen and colleagues [25] reported a survival rate of 45% after 24 h and 25% after 96 h using the same microorganism at a lower dose of 10^6^ CFU/larvae. These discrepancies are often due to non-unified experimental protocols, whether in terms of rearing and subject maintenance techniques or infection methods. Specifically, for bacterial infection by injection, the volume of the injection can also influence the strength of cellular and humoral immune responses, as a larger dose may provoke a stronger immune reaction [38]; furthermore, the literature highlights the importance of a well-defined infection pattern with *G. mellonella*, emphasizing the need to carefully consider methodological variables in the experimental design [39]. 

The standardized infection protocol described in this paper aims to provide a reproducible method for studying Gram-positive bacterial infections using an alternative animal model. This approach seeks to address issues encountered in the scientific literature while making invertebrate treatments standardized and reproducible for broader adoption in the scientific community. A deeper understanding of the interaction between *M. luteus* and the immune system of *G. mellonella* may offer valuable insights into the mechanisms of bacterial virulence and host defenses. 

The innate immune system of insects, while comparable in some processes to that of higher animals [29,40], differs significantly as it lacks B lymphocytes and antibodies. Consequently, adaptive immunity processes cannot be studied using these models. Despite these limitations, *G. mellonella* larvae provide a robust and ethical alternative to vertebrate models for toxicological and immunological studies.

Furthermore, the specific conditions under which the larvae are maintained and fed, such as temperature and humidity, can significantly influence experimental outcomes and must be meticulously controlled [41,42] and standardized. Susceptibility to infection can be affected by various factors, including genetic differences and rearing techniques, such as environmental parameters. For instance, Mowlds and colleagues described the impact of heat conditioning prior to testing, demonstrating that heat shock induces the activation of immune processes and alters the basal physiological state of the larvae [43].

Despite these considerations, bacterial pathogenesis studied in *G. mellonella* has yielded results comparable to those obtained in mammalian models [44]. Therefore, it is essential for researchers to establish standardized protocols and carefully control experimental variables to ensure the reproducibility of their studies.

## 5. Conclusions

The present study aimed to establish an infection protocol for the Gram-positive microorganism *M. luteus* in *G. mellonella*. This model can be used for further investigations into the role of the insect immune system in bacterial clearance and as an experimental platform for the preclinical study of new antibiotics. To obtain reliable and reproducible results, it is essential to ensure a complete genetic characterization of the larvae and to source them from certified suppliers.

## Figures and Tables

**Table 1:** Summary of the main experimental procedures.

*G. mellonella* breeding and maintenance	Larvae fed standard diets and maintained in climatic chambers, under controlled conditions of temperature and relative humidity RH (26 °C, RH 60%, in the dark). Last stage (300–350 mg) healthy larvae were selected for the assays.
Definition of bacterial growth curves	Choice of the bacterial species and strain. Bacterial growth on LB, overnight at optimal growth temperature under shaking. Streaking on solid medium plates, incubated at optimal growth temperature overnight. Single colony inoculum on liquid medium, monitoring for 24 h under shaking. Hourly control of growth curve by turbidimetry (OD_600_) correlated with colony count by track dilution assay.
*G. mellonella* larvae infection	Sterilization (70% ethanol) and anesthesia by cooling on ice. Infection with bacterial doses (CFU/larvae) by injection into the abdominal spiracle area, with a gas-tight micro-syringe. Injected larvae incubated at 37 °C in the dark, in climatic chamber up to 7 days.
*G. mellonella* survival assessment	Control of survival over time by visual testing, based on morphological changes (darkening) and motility of larvae. Definition of LD_50_ and Kaplan–Meier survival curves. Course of infection by CFU counts in hemolymph samples.

**Table 2:** Average OD and relative SD over 24 h, (n = 10).

Time (h)	Avg. OD_600_ ± SD	Time (h)	Avg. OD_600_ ± SD
1	0.01516 ± 0.00497	13	0.6695 ± 0.1306
2	0.08917 ± 0.0566	14	0.6970 ± 0.1396
3	0.1914 ± 0.0709	15	0.6955 ± 0.1320
4	0.2519 ± 0.06887	16	0.7351 ± 0.1236
5	0.3269 ± 0.0552	17	0.7481 ± 0.1163
6	0.3810 ± 0.04774	18	0.7621 ± 0.1122
7	0.4152 ± 0.05886	19	0.7682 ± 0.07287
8	0.4701± 0.08039	20	0.7768 ± 0.0704
9	0.5109 ± 0.08614	21	0.7599 ± 0.06782
10	0.5604 ± 0.09189	22	0.7537 ± 0.04511
11	0.5823 ± 0.08206	23	0.7474 ± 0.05197
12	0.6180 ± 08842	24	0.7469 ± 0.04244

**Table 3:** Average CFU/mL and relative SD over 24 h, (n = 5).

Time (h)	Avg. CFU/mL ± SD	Time (h)	Avg. CFU/mL ± SD
1	5.67 × 10^7^ ± 5.77 × 10^6^	13	4.80 × 10^8^ ± 5.83 × 10^7^
2	8.00 × 10^7^ ± 1.00 × 10^7^	14	5.14 × 10^8^ ± 1.31 × 10^8^
3	1.98 × 10^8^ ± 8.73 × 10^7^	15	4.64 × 10^8^ ± 1.17 × 10^8^
4	3.20 × 10^8^ ± 5.35 × 10^7^	16	5.12 × 10^8^ ± 1.36 × 10^8^
5	4.40 × 10^8^ ± 1.01 × 10^8^	17	6.20 × 10^8^ ± 2.20 × 10^8^
6	4.28 × 10^8^ ± 1.24 × 10^8^	18	4.80 × 10^8^ ± 1.88 × 10^8^
7	5.20 × 10^8^ ± 1.87 × 10^8^	19	6.03 × 10^8^ ± 9.83 × 10^7^
8	5.70 × 10^8^ ± 5.83 × 10^7^	20	4.95 × 10^8^ ± 1.10 × 10^8^
9	5.55 × 10^8^ ± 2.52 × 10^7^	21	4.58 × 10^8^ ± 8.16 × 10^7^
10	5.45 × 10^8^ ± 4.20 × 10^7^	22	5.60 × 10^8^ ± 3.00 × 10^7^
11	5.47 × 10^8^ ± 9.07 × 10^7^	23	5.00 × 10^8^ ± 1.73 × 10^7^
12	4.60 × 10^8^ ±1.43 × 10^8^	24	4.85 × 10^8^ ± 6.81 × 10^7^

## Data Availability

The data presented in this study are available upon request to the corresponding author.
